# Identification of Novel Targets of CSL-Dependent Notch Signaling in Hematopoiesis

**DOI:** 10.1371/journal.pone.0020022

**Published:** 2011-05-26

**Authors:** Habib Hamidi, Derek Gustafason, Matteo Pellegrini, Judith Gasson

**Affiliations:** 1 Department of Biological Chemistry, David Geffen School of Medicine, University of California Los Angeles, Los Angeles, California, United States of America; 2 UCLA-DOE Institute for Genomics and Proteomics, University of California Los Angeles, Los Angeles, California, United States of America; 3 Department of Molecular, Cell, and Developmental Biology, University of California Los Angeles, Los Angeles, California, United States of America; 4 Division of Hematology-Oncology, Department of Medicine, and Department of Biological Chemistry and Jonsson Comprehensive Cancer Center, David Geffen School of Medicine, University of California Los Angeles, Los Angeles, California, United States of America; Instituto Nacional de Câncer, Brazil

## Abstract

Somatic activating mutations in the Notch1 receptor result in the overexpression of activated Notch1, which can be tumorigenic. The goal of this study is to understand the molecular mechanisms underlying the phenotypic changes caused by the overexpression of ligand independent Notch 1 by using a tetracycline inducible promoter in an *in vitro* embryonic stem (ES) cells/OP9 stromal cells coculture system, recapitulating normal hematopoiesis. First, an *in silico* analysis of the promoters of Notch regulated genes (previously determined by microarray analysis) revealed that the motifs recognized by regulatory proteins known to mediate hematopoiesis were overrepresented. Notch 1 does not bind DNA but instead binds the CSL transcription factor to regulate gene expression. The *in silico* analysis also showed that there were putative CSL binding sites observed in the promoters of 28 out of 148 genes. A custom ChIP-chip array was used to assess the occupancy of CSL in the promoter regions of the Notch1 regulated genes *in vivo* and showed that 61 genes were bound by activated Notch responsive CSL. Then, comprehensive mapping of the CSL binding sites genome-wide using ChIP-seq analysis revealed that over 10,000 genes were bound within 10 kb of the TSS (transcription start site). The majority of the targets discovered by ChIP-seq belong to pathways that have been shown by others to crosstalk with Notch signaling. Finally, 83 miRNAs were significantly differentially expressed by greater than 1.5-fold during the course of *in vitro* hematopoiesis. Thirty one miRNA were up-regulated and fifty two were down-regulated. Overexpression of Notch1 altered this pattern of expression of microRNA: six miRNAs were up-regulated and four were down regulated as a result of activated Notch1 overexpression during the course of hematopoiesis. Time course analysis of hematopoietic development revealed that cells with Notch 1 overexpression mimic miRNA expression of cells in a less mature stage, which is consistent with our previous biological characterization.

## Introduction

Notch proteins are single-pass, heterodimeric, transmembrane proteins encoded by genes which are conserved from flies to humans. Notch plays a critical role in development mediated by cell-cell interaction. Upon binding of a ligand (a single pass transmembrane protein on a neighboring cell) the Notch receptor undergoes a series of proteolytic cleavages resulting in the release of the Notch intracellular domain (NICD). The NICD translocates to the nucleus and activates the transcription of target genes by turning the CSL transcription factor from a repressor to an activator [1] (reviewed in Kopan et al.).

Aberrant Notch signaling has been associated with many cancers including leukemia [2], breast cancer [3], medulloblastoma [4], melanoma [5] and pancreatic cancer [6] . In some reports it has been described as tumorigenic while in other reports it's been described as having tumor suppressor function. In leukemia the discovery of the (7;9) chromosomal translocation [7] showed that constitutively active Notch signaling can be tumorigenic. Although the translocation was later found in less than 1% of T-ALL, somatic activating mutations in Notch1 receptor were detected in over 50% of human T-ALL cases [2] and 74% of tumors in a mouse leukemia model [8], showing that overexpression of activated Notch1 is indeed tumorigenic [9].

One possible mechanism of oncogenesis is the disruption of CSL binding homeostasis. An abundance of NICD has been shown to stoichiometrically deplete CSL from other binding partners and their associated genomic loci leading to aberrant gene regulation at those sites (10). CSL can associate with at least one partner other than Notch, p48/PTF1a [10], [11], [12]. This disruption may lead to altered gene regulation of target genes that are important in regulating growth. A genome wide assessment of CSL in the mammalian genome has not yet been performed to assess which genes are regulated by CSL.

Furthermore, it has been demonstrated that Notch signaling is context dependent in cancer, based on its integration with other signaling pathways. The Notch pathway has been shown to crosstalk with Wnt, Cadherin and the Sonic Hedgehog pathways which have been associated with tumor formation in a variety of cancers. When Notch was activated at different stages of mesodermal differentiation, the majority of the genes regulated by Notch1 were cell type specific and dependent on the other signals [13]. We wanted to assess CSL binding sites globally to examine if they are found in the regulatory region of genes mediating important signaling pathways and if CSL binding sites are distributed throughout the genome indicating that Notch signaling is integrating with signaling pathways at the level of transcription.

The molecular mechanism underlying the function of Notch1 in disease and developmental states has been investigated by identification of either the direct targets of Notch1 or the direct targets of the effector protein of Notch1 signaling CSL. An integrated systems biology approach was used to assess the direct targets of Notch1 during leukemic cell growth [14]. First, differentially regulated genes were determined using microarray analysis comparing gene expression profiles of seven T-ALL cell lines treated with either DMSO or a highly active gamma secretase inhibitor. This was followed by identifying the direct targets of Notch1 in the HPB-ALL T-ALL cell line using a ChIP on chip (ChIP-chip) analysis using a spotted promoter array platform. Although the microarray analysis identified differential expression of several known direct targets of Notch signaling their ChIP-Chip analysis could not confirm promoter occupancy of Deltex1, Hes1 nor Notch3 by NICD. This may be a reflection of the array platform which only included the proximal promoter regions (−700 to +200 bp). For example, only 18% of MYC-binding sites were found to be within 1 kb of a 5′-exon using an oligonucleotide tiling array that encompasses chromosomes 21 and 22 [15]. Any binding site outside of the core promoter regions would be missed by this analysis which may include many well defined targets of Notch signaling.

Notch 1 does not bind DNA and therefore assessing the occupancy of Notch1 at the promoters of target genes may be limited by the technical difficulty of the Notch 1 antibody IP. The Bray group sought to assess the direct targets of Notch signaling by assessing the promoter occupancy of the Notch 1 effector protein CSL in Drosophilia DMD8 cells [16] . The DMD8 cell model was used to assess global changes in mRNA expression (microarray analysis) and genome wide occupancy of CSL (Su(H) in *Drosophila*) within 30 min of activating Notch using ChIP-chip analysis in hopes of identifying direct target of CSL dependent Notch signaling. Although, their genome wide promoter occupancy analysis benefited from the use of an array that tiled the *Drosophila* genome to give a more complete assessment of Notch target genes, it still suffers from the general limitation of ChIP-chip technology including probe selection bias and hybridization bias. Only 262 significant Su(H) binding peaks were identified genome wide. Computational analysis of the tiling array was based on a method to detect peaks in a dense tiling array which included tiling one 50-mer every 38 bp [17] . However, the array used by the Bray group included 60 base oligonucleotide probes printed for approximately every 300 bp of the genomic DNA and thus it would likely miss true positive because of the limitation of the array. A significant peak was defined as a region that was detected in five adjacent probes which corresponds to 1.5 kb region. Even though, there was a statistical enrichment of Su(H) sites in the peaks they identified, only 27% of the binding-site clusters identified computationally and 1.08% of high scoring Su(H) binding sites in noncoding regions were identified as occupied.

Assessing transcription factor binding sites genome-wide has only become possible the last few years. High-throughput sequencing combined with Chromatin Immunoprecipitation has become the gold standard for assessing transcription factor binding sights globally *in vivo*. It is preferred over ChIP-chip because it is an absolute rather than a relative assessment of the genomic loci bound by the protein of interest. With ChIP-seq you actually sequence the ChIP purified DNA instead of hybridizing it to set of preselected probes.

Genome wide occupancy of CSL will also be important in assessing if Notch is regulating microRNAs which are important regulators of development. MicroRNAs (miRNA are short (19–25 nucleotides in lenght) noncoding RNAs, that regulate gene expression by either inhibiting translation or marking specific mRNA for degradation [18], [19]. MiRNAs influence gene expression as broadly as transcription factors and have been shown to play a role in regulating development [19]. MiRNA target predictions have indicated that miRNA may target nearly 30% of animal genes [20], [21], [22]. Thus, its not surprising that perturbation in their homeostatic function has been associated with many diseases including cancer [23], [24], [25]. The miR-15a and miR-16-1 genes target B cell lymphoma 2 (Bcl2), an antiapoptotic gene and thus loss of their expression has been associated with cancer [26], [27]. Some miRNAs such at the miR17–92 locus 13q31 were shown to have oncogenic potential because they are amplified in some tumor [28] and their overexpression in a mouse model actually accelerated tumorigenesis [28], [29].

MiRNAs play an important role in regulating hematopoiesis [30]. MiR-142 was highly expressed in all hematopoietic tissues whereas miR-223 was expressed exclusively in the bone marrow which consists of hematopoietic stem cells and myeloid, erythroid and lymphoid cells at different stages [30]. MiR-223 is also associated with myeloid differentiation [31]. Other groups have also implicated miR-144, miR-150, and miR-155 in hematopoiesis [32]. MiR-126 has been associated with megakaryocyte differentiation [33]. The miR-144/451 cluster is upregulated during erythropoiesis and is under the control of the master erythrocyte regulator GATA-1 [34], [35].

The crosstalk between microRNA expression and Notch signaling hasbeen reported in terms of which microRNAs target the Notch pathway or its target genes. Mir-34a has been reported to downregulate Notch-1 Notch-2, and CDK6 protein expression [36]. MiR-1 negatively regulates Delta-1 protein levels in mouse embryonic stem cells [37]. MiR-1995b down regulates the down stream Notch target gene Hes-1 [38]. These miRNA have been studied as potential therapeutic targets for cancer. However, in leukemia, its not abberrent gene regulation that leads to constititutively active Notch-1 expression, its somatic activating mutations in the receptor which allows the receptor to have increase stability. Therefore, finding miRNAs that are regulated by Notch signaling may be another potential therapeutic target.

CSL is a unique transcription factor because it is bound regardless of the presence of activated Notch. A conventional ChIP with antibodies to CSL alone would be limiting because the effect of Notch signaling would not be gauged. Furthermore, it would be difficult to discern real CSL binding sites from artifacts. A sequential ChIP is a new method that allows one to assess transcription factor binding occupancy using two IgGs. A hallmark of Notch activation is the acetylation of H4. Thus, a sequential ChIP with antibody to acetylated H4 followed by antibody against CSL will identify activated Notch responsive CSL binding sites. Furthermore, CSL is a small protein 60 kDa and bound to DNA in the presence of either repressor complexes or activation complexes which are enormous in size. A sequential ChIP, especially since the first IP is against the readily accessible acetylated H4 would help reduce the complexity of the nuclear lysate to allow for optimal IP with CSL antibody in the second IP. This will possibly overcome the technical difficult associated with performing a ChIP against CSL.

Embryonic stem (ES) cells have been valuable for understanding the biology of tissue development and may serve as potential therapy for diseases such as cancer. The study of the murine hematopoietic system has resulted in the major technological advances in deriving mature tissues from embryonic stem (ES) cells [39] and its further characterization will have implications beyond the study of the blood system. The induction of hematopoietic differentiation on stromal cells [40] and formation of embryoid bodies (EB) [41], [42] are the two experimental systems used to generate hematopoietic precursors from embryonic stem cells in most experiments [43]. We have previously modeled murine hematopoiesis using an embryonic stem cells (ES)/OP9 coculture which was shown to be a highly reproducible way to model hematopoiesis in vitro [40], [44], [45], [46]. The OP9 stroma cell line provides the necessary extrinsic signals for the differentiation of pluripotent ES cells first into primitive flk1+ hemangioblasts (day 4–5) and then immature hematopoietic stem and progenitor cells (day 8). We have shown that the overexpression of ligand independent Notch1 in flk1+ hemangioblasts results in an alteration of the phenotype of the day 8 hematopoietic progenitor cells characterized by cell morphology, flow cytometry and gene expression profiling.

The goal of this study is to understand the molecular mechanism underlying the phenotypic changes caused by the expression of ligand independent Notch 1. First, we performed an *in silico* analysis of the promoters of 148 previously identified Notch regulated genes to determine the presence of putative regulatory regions. Then, a custom ChIP-chip array was used to assess the occupancy of CSL in the promoter regions of these 148 differentially expressed genes. Finally, a comprehensive mapping of the CSL binding sites genome-wide was determined using ChIP-seq analysis. Given that miRNA have documented roles in hematopoietic development, we wanted to assess which miRNAs are regulated during normal hematopoiesis and which miRNA are differentially regulated by overexpression of Notch. Thus, we performed expression profile analysis of microRNAs, using microRNA microarray during normal hematopoiesis and in response to overexpression of ligand independent Notch1.

## Results

### Identification of putative regulatory motifs in the upstream regions of differentially expressed genes

Our lab previously utilized an *in vitro* murine embryonic stem cell co-culture system to study the effects of activated Notch 1 on normal hematopoietic differentiation. A tetracycline-inducible system regulating expression of a ligand independent, constitutively active form of Notch1 was introduced into murine E14Tg2a ES cells. The ES cells were co-cultured with OP9 stromal cells to induce the ES cells to differentiate first to hemangioblasts and subsequently to hematopoietic progenitors. During days 5 to 8 of the co-culture flk1+ hemangioblasts develop into hematopoietic progenitors, which then go on to form mature myeloid and erythroid cells. Previously we showed that overexpression of ligand independent Notch1(Notch On), a phenotype mimicking the abnormal expression of Notch1 in leukemia [9] , leads to a distinct phenotype that can be characterized by flow cytometry analysis (over-expression of Notch 1 preserves cells in a less mature state) and gene expression profiling [45].

Global gene expression profiling of day 8 hematopoietic progenitors in the absence and presence of activated Notch yielded 158 differentially-regulated candidate genes [45] as both direct and indirect putative downstream targets of oncogenic forms of Notch. The Panther Database system [47] was used to identify Gene Ontology (GO) Biological Process categories for the 158 differentially regulated genes to identify the pathways regulated by Notch. Not surprisingly eight of the genes mapped to the Notch pathway (Table 1). Although only the Notch, Angiogenesis and Alzheimers disease-presenilin pathways were significantly over-represented (P-value<0.01), the Panther analysis showed that the 68 classified genes represented multiple important signaling pathways such as the Wnt, Cadherin, TGFbeta and Integrin pathways, all known to be important signaling pathways in development. Of the 158 genes, 90 were categorized as unclassified.

**Table 1:** Gene Ontology of differentially regulated genes.

	NCBI:Mm	Microarray		
Pathways	#	#	Expected	P value
Unclassified	26616	90	116.55	5.84E-08
Notch signaling pathway	52	8	0.23	1.98E-08
Angiogenesis	258	7	1.13	0.0259
Alzheimer disease-presenilin pathway	143	6	0.63	0.00743
Integrin signaling pathway	263	6	1.15	0.188
Wnt signaling pathway	408	5	1.79	1
TGF-beta signaling pathway	154	4	0.67	0.813
Cadherin signaling pathway	204	3	0.89	1
Apoptosis signaling pathway	187	3	0.82	1
Insulin/IGF pathway-protein kinase B signaling cascade	89	2	0.39	1
Insulin/IGF pathway-MAP kinase cascade	77	2	0.34	1
Blood Coagulation	69	2	0.3	1
p38 MAPK pathway	61	1	0.27	1
p53 pathway	135	1	0.59	1
T cell activation	168	1	0.74	1
Nicotine acetylcholine receptor signaling pathway	104	1	0.46	1
Muscarinic acetylcholine receptor 2 and 4 signaling pathway	63	1	0.28	1
Arginine biosynthesis	6	1	0.03	1
Interleukin signaling pathway	169	1	0.74	1
Inflammation by chemokine and cytokine signaling pathway	337	1	1.48	1
FAS signaling pathway	45	1	0.2	1
Endothelin signaling pathway	97	1	0.42	1
EGF receptor signaling pathway	153	1	0.67	1
Cytoskeletal regulation by Rho GTPase	135	1	0.59	1
Axon guidance mediated by netrin	42	1	0.18	1

To further assess which factors may be involved in or impacted by the differential regulation of the 158 genes we performed an *in silico* analysis of their promoter regions. CLOVER is an algorithm developed by Frith et al. to identify transcription factor binding sites that are statistically over- or under-represented in a group of sequences. We used CLOVER to assess whether the binding sites of known regulatory proteins compiled in the JASPAR library were over or under-represented in the set of sequences representing 1.5 kb upstream of the promoters of 148 of the 158 regulated genes. The results showed that there were 13 motifs, including the CSL motif, that were over-represented and 2 motifs that were under-represented in the 158 differentially expressed genes (Table 2).

**Table 2:** Significant JASPAR motifs over and under represented within 1.5 kb upstream of TSS of Notch regulated genes.

ChipSeq Peaks	DNA Binding Site (TFBS)	TF	Gene ID	Function
	Over-Represented (p-value <0.01)			
1	CSL Binding Motif	CSL	NM_009035	Mediator of Notch Signaling
1	Gklf ZN-FINGER, C2H2	Klf4	U20344	Hematopoiesis (Earyl Erythropoiesis) [48]
1	Pax-4 PAIRED-HOMEO	Pax4	AF031150	Different. of pancreatic islet beta [49]
1	BC 1-4 ZN-FINGER, C2H2	Kbtbd4	NM_025991	N/A
1	HNF-3beta FORKHEAD	Foxa2	NM_010446	Early Embryogensis [50]
1	MZF_1-4 ZN-FINGER, C2H2	Zfp98	NM_016793	Hematopoiesis (Myelopoiesis) [51]
1	MEF2 MADS	Mef2a	AV255689	Myogenesis [52]
0	Athb-1 HOMEO-ZIP	Hhex	AK014111	Hematopoiesis [53]
1	Nkx HOMEO	Nkx2-5	NM_008700	Cardiac development [54], leukemogenesis [55]
0	NF-Y CAAT-BOX	Nfya	NM_010913	Promotes HSC self-renewal [56]
0	SRY HMG	Sox3	NM_009237	Neuronal Development [57]
1	HNF-1 HOMEO	Tcf1/HNF1	NM_009327	Pancreatic Development [58]
1	cEBP bZIP	Cebpg	BC011319	Hematopoiesis (Myelopoiesis) [59]
	Under-Represented (p-value <0.01)			
1	Yin-Yang ZN-FINGER, C2H2	Yy1	NM_009537	myeloid transforming gene [60]
1	GATA-2 ZN-FINGER, GATA	Gata2	NM_008090	hematopoietic differentiation[61]

Twelve out of thirteen regulatory proteins recognizing the over-represented motifs have previously been shown to have a role in stem cell differentiation while 8/13 (KLF4, Nkx2.5, Zfp98, Fox2a, CSL, Hhex, Nfya, Cebpg) have been shown to have a role in hematopoiesis specifically. Furthermore, the two underrepresented motifs (Gata2 and Yy1) also play critical roles in hematopoietic development. It seems likely that they are under-represented in this experiment because they play a role in the later steps of hematopoiesis whereas activated Notch 1 signaling preserves the cells in a less differentiated state.

The microarray data were searched to determine if the genes encoding the transcription factors associated with the 13 over-represented and 2 under-represented motifs were transcribed in the cells and if they were differentially regulated by activated Notch. 9/13 transcription factors whose TFBS were over-represented had mRNA levels higher than the arbitrary cutoff of 500 (Table 3). Both of the transcription factors that were under-represented also had mRNA levels higher than 500. However, only KLF4 was differentially regulated by Notch showing an increase in expression of almost 3 fold.

**Table 3:** Regulatory proteins are not transcriptionally regulated by Notch.

			Microarray Fold Change		mRNA Levels	
DNA Binding Site	Associated Protein	Gene ID	ON/OFF	SD	OFF	ON
Over-Represented (p-value<0.01)						
CSL Binding motif	CSL	NM_009035	0.8	0.2	2803.4	2179.6
Gkfl ZN-FINGER, C2H2	Klf4	U20344	**2.8**	1.4	1582.2	4932
Pax-4 PAIRED-HOMEO	Pax4	AF031150	2	1.1	17.6	35.1
Broad-complex1-4 ZN-FINGER	Kbtbd4	NM_025991	1	0	2188.4	2405.5
HNF-3beta FORKHEAD	Foxa2	NM_010446	1.5	1.1	382.3	458.3
MZF_1-4ZN-FINGER, C2H2	Zfp98	NM_016793	1.8	1	41.2	63.3
MEF2 MADS	Mef2a	AV255689	1	0.1	4377.5	4940
Athb- 1 HOMEO-ZIP	Hhex	AK014111	0.7	0.1	8542.9	6399.7
Nkx HOMEO	Nkx2.5	NM_008700	1.2	0.4	528.6	648
NF-Y CAAT-BOX	Nfya	NM_010913	0.7	0	1351.6	1077
SRY HMG	Sox3	AF434675	0.6	0.4	148.5	52
HNF-1 HOMEO	Tcf1/HNF1	NM_009237	4.2	4.1	111.2	277
cEBP bZIP	Cebpg	BC011319	1.2	0.4	494	596.5
Under-represented (p-value>0.99)						
Yin Yang ZN-FINGER, C2H2	Yy1		0.9	0.1	11616	11426.3
GATA-2 ZN-FINGER, GATA	Gata2		1	0.1	2651.4	2815.4

### Occupancy of CSL binding sites in the upstream regions of differentially expressed genes

Twenty eight of the 148 Notch regulated genes had a putative CSL Binding Site within their promoter (defined as the sequence 1.5 kb upstream of their transcriptional start site) (TSS) (Table 4). The list includes known targets of Notch signaling such as Hey1, Hes1 and Notch 1 (shown in the bold type) along with a cohort of novel targets including wnt4. Gene ontology analysis (data not shown) indicates that known targets of Notch signaling are enriched along with the presenilin processing pathway. Members of the Wnt, Integrin and Cadherin pathways are present among the list.

**Table 4:** Notch regulated genes with a putative CSL Binding Site within 1.5 kb upstream of TSS.

Microarray Fold Change	Genbank	GENE_SYMBOL
56.27	NM_008570	MCPT1
47.23	NM_008182	GSTA2
***22.97***	***NM_010423***	***HEY1***
12.27	NM_008182	MCPT1
*6.11*	*NM_013749*	*TNFRSF12A*
5.04	NM_009523	WNT4
*4.66*	*NM_010664*	*KRT18*
*4.52*	*NM_013464*	*AHR*
*4.02*	*NM_013749*	*TNFRSF12A*
3.88	BC004651	GM2A
**3.84**	**NM_008714**	**NOTCH1**
3.69	NM_021334	ITGAX
3.34	NM_010299	GM2A
3.34	U03561	HSPB1
3.33	BC013560	COL4A2
3.11	NM_080858	ASB12
*3.08*	*NM_008086*	*GAS1*
3.08	AK019971	PRRX2
*2.98*	*NM_134163*	*MBNL3*
**2.80**	**NM_013905**	**HEYL**
*2.40*	*AF017174*	*CPT1B*
*2.36*	*NM_013560*	*HSPB1*
2.26	NM_011658	TWIST1
2.17	BC025600	TMEM119
***2.13***	***BC018375***	***HES1***
**1.91**	**NM_008716**	**NOTCH3**
**1.71**	**NM_008716**	**NOTCH3**
1.71	D45203	D0H4S114
0.46	NM_008625	MRC1
*0.38*	*BC005440*	*PTGER2*
0.35	BI110565	POSTN

Genes confirmed by ChIPseq in italic.

A modified ChIP procedure was used to assess CSL occupancy in the upstream region of of Notch regulated genes *in vivo*. Two major modifications were made to the ChIP procedure in these experiments. Notch is thought to modify the function of CSL and not it's binding to the DNA; association of the Notch ICD with bound CSL protein changes its function from a repressor to an activator. Therefore, we added an extra IP step to the standard ChIP procedure to assess CSL occupancy within activated promoters using an antibody that recognizes acetylated Histone H4. Acetylation of both Histone H3 and H4 is a hallmark of transcriptional activation [62], [63], mediated in part by p300, a co-activator that is a component of the NICD:CSL transcriptional activation complex [64]. Using this 2 step IP ChIP procedure (Re-ChIP) activated Notch responsive CSL occupancy was identified.

For the second modification to the standard ChIP protocol, ChIP purified DNA fragments were hybridized to a custom high density oligonucleotide array which consisted of tiled, 50 bp probes constituting 1.5 kb of DNA sequence upstream of the transcriptional transcription start site (TSS) of 148 of the 158 genes identified by microarray analysis (sequences were not available for the remaining 10 genes). DNA isolated from the two-step ChIP procedure (Re-ChIP)using cells exposed to activated Notch (Notch On) and control cells (Notch Off) were hybridized to this custom array. Experimental conditions were the same as those used to isolate the RNA and generate the list of 158 Notch regulated genes (original microarray analysis). Since each probe was present at least twice, a quality assessment of the in-array variability was controlled by only including probes with a CV of less than 30%.

Activated Notch responsive CSL binding was determined using the ChIPOTLE algorithm to identify peaks using the Notch On/Notch Off signal from the ChIP-chip. The ChIPOTLe algorithm has been used to analyze yeast ChIP-chip data generated on whole-genome tiling arrays^33^ The Gaussian distribution was used to model the background and P value of 0.01(corrected for multiple testing). The results show that of the 148 tested genes, 61 genes were bound by activated Notch responsive CSL (Table 5).

**Table 5:** Differentially regulated genes shown by Chip-ChIP to be bound by activated Notch responsive CSL.

Affy	P-value	Foldchange	Common	Genbank
1418403_at	0.0368	3.665	Adam19	NM_009616
1425405_a_at	0.0415	0.471	Adar	AF291876
1421480_a_at	0.0448	1.981	Adarb1	NM_130895
1422514_at	0.0368	2.356	Aebp1	NM_009636
1418204_s_at	0.0495	5.204	Aif1	NM_019467
1449027_at	0.0319	2.351	Arhu	NM_133955
1416239_at	0.0277	1.828	Ass1	NM_007494
1419406_a_at	0.012	1.751	Bcl11a	NM_016707
1417381_at	0.0484	3.723	C1qa	NM_007572
1450355_a_at	0.046	0.556	Capg	NM_007599
1448261_at	0.03	3.603	Cdh1	NM_009864
1424051_at	0.0229	3.327	Col4a2	BC013560
1417014_at	0.0435	2.993	Cryac	AF250139
1418365_at	0.0011	3.047	Ctsh	NM_007801
1448591_at	0.0191	2.227	Ctss	NM_021281
1450839_at	0.0177	1.71	D0H4S114	D45203
1434348_at	0.0114	0.553	D17Ertd315e	BM206792
1434442_at	0.0025	2.021	D5Ertd593e	BB667844
1449222_at	0.0236	0.475	Ebi3	NM_015766
1416552_at	0.0142	4.126	Esg1	NM_025274
1418572_x_at	0.0401	4.021	Fn14-pending	NM_013749
1416855_at	0.0283	3.085	Gas1	NM_008086
1418949_at	0.0491	2.314	Gdf15	NM_011819
1421040_a_at	0.0187	47.23	Gsta2	NM_008182
1418102_at	0.0413	2.126	Hes1	BC018375
1415999_at	0.0014	22.973	Hey1	NM_010423
1422943_a_at	0.0289	2.364	Hsp25	NM_013560
1416630_at	0.0102	2.621	Idb3	NM_008321
1426858_at	0.0201	3.048	Inhbb	BB353211
1450029_s_at	0.0263	2.314	Itga9	BG067332
1416401_at	0.0033	2.509	Kai1	NM_007656
1448169_at	0.0476	4.659	Krt1-18	NM_010664
1448237_x_at	0.0024	1.711	Ldh2	NM_008492
1451344_at	0.0449	2.172	MGC38046	BC025600
1419402_at	0.0228	1.696	Mns1	NM_008613
1450430_at	0.023	0.456	Mrc1	NM_008625
1448990_a_at	0.0487	1.599	Myo1b	AI255256
1454903_at	0.0067	27.008	Ngfr	BB151515
1418633_at	0.0058	3.842	Notch1	NM_008714
1421965_s_at	0.009	1.906	Notch3	NM_008716
1449146_at	0.0162	4.568	Notch4	NM_010929
1417986_at	0.0025	5.89	Nrarp	BI696369
1423606_at	0.0111	0.35	Osf2-pending	BI110565
1436970_a_at	0.0077	2.979	Pdgfrb	AA499047
1416321_s_at	0.0439	2.689	Prelp	BC019775
1420664_s_at	0.0127	2.544	Procr	NM_011171
1424704_at	0.0386	3.184	Runx2	D14636
1419480_at	0.0167	0.389	Sell	NM_011346
1423129_at	0.0158	0.486	Shoc2	BQ032685
1460292_a_at	0.0018	6.996	Smarca1	NM_053123
1455900_x_at	0.011	2.803	Tgm2	BB041811
1418726_a_at	0.0225	4.032	Tnnt2	NM_011619
1450782_at	0.0088	5.045	Wnt4	NM_009523
1436791_at	0.0062	5.878	Wnt5a	BB067079
1421498_a_at	0.0258	4.111	0610010M13Rik	NM_023450
1452747_at	0.0338	0.58	1110012E06Rik	BM944122
1438511_a_at	0.0348	3.541	1190002H23Rik	BB408123
1420336_at	0.0262	2.093	2010109H09Rik	NM_025629
1424770_at	0.0021	1.798	4833423D12Rik	BI248947
1418776_at	0.0248	4.486	5830443L24Rik	NM_029509

As a control a single IP ChIP with only CSL was analyzed under the same parameters and as expected there were no peaks detected when comparing experimental to control conditions. This result is expected as CSL is thought to be bound to its TFBS in both the presence and absence of Notch. In the presence of Notch the complex is converted from a repressor to an activator recognized by the acetylated H4 antibody.

### Genome wide mapping of CSL binding sites during hematopoiesis

To generate genome wide maps of CSL binding *in vivo*, Re-ChIP purified DNA fragments were isolated from control cells and cells with activated Notch1 overexpressed from Day 5 to Day 8 as previously reported. The experimental condition were the same as as those used to generate Re-ChIP fragments for the ChIP-chip analysis and to isolate the RNA for the original microarray analysis.

**Figure 1:**
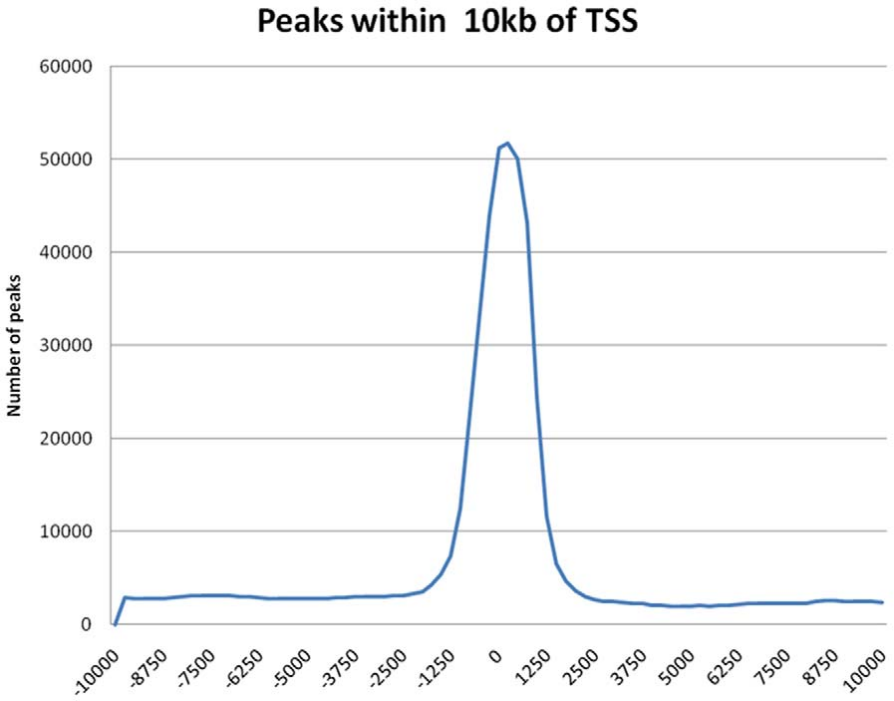
Most unique reads map within 1.5 kb of the transcriptional start site. Uniquely-aligned sequences (reads) were counted within a given 1000 base window relative to genomic positions. A step size of 50 bases was used for window overlap. Poisson distribution [116] was then used to generate P-values for each 1000 base window and windows were filtered by these values to generate a list of peaks. Significant peaks were windows with P-value less than 10^−12^.

These ChIP purified DNA fragments were then sequenced using massive parallel sequencing instead of hydbridizing them to an array. An experiment sequenced in technical triplicates resulted in 36 base pair (bp) sequence reads which were aligned to the reference mouse genome. 72.9% of the Notch On and 83.9% of the Notch Off uniquely mapped reads aligned to the genome with zero-mismatches. Figure 1 shows that the unique reads mapped preferentially to regions within 1.5 kb from the transcriptional start sites indicating that the Re-ChIP DNA fragments are enriched in the proximal promoter of genes instead of randomly distributed.

**Figure 2:**
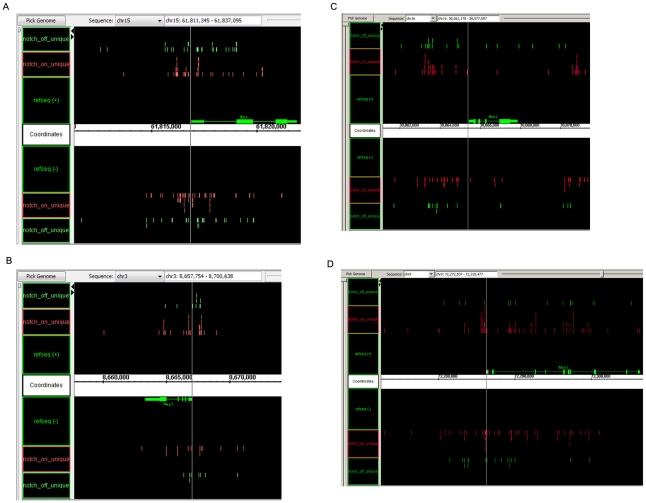
ChIP-seq reads mapping near the TSS. The Integrated Genome Browser was used to visualize the ChIPseq peaks from both the Notch On and Notch Off sample. (a) TSS of the Myc gene. (b) TSS of the Hey1 gene (c) Hes1 gene and (d) Mns1 gene.

The unique reads were used to identify regions of the genome with significant enrichment in CSL associated DNA sequenced using a peak finding algorithm. A peak was defined as a 1000 bp region with a P-value less than 10^−12^ along with a window mapability of greater than 25% and sense/antisense strand count within 30%. Screenshots of ChIP-seq reads using the genome browser show that ChIP-seq reads map upstream of known targets of Notch such as the Myc [65]oncogene (Figure 2a), Hey1 [66] (Figure 2b) and Hes1 [66](Figure 2c) as well as novel targets such as Mns1(Figure 2d).

**Figure 3:**
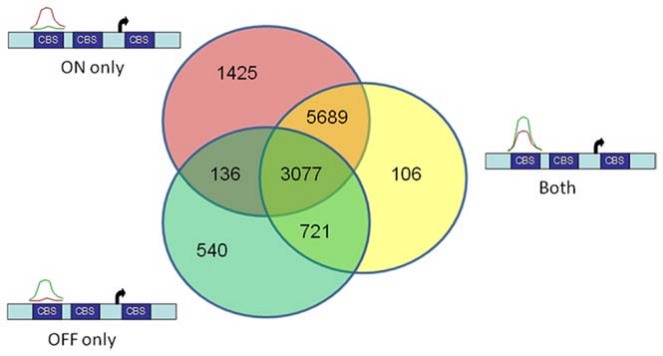
Number of genes with at least one significant peak within 10 kb of the TSS. Venn diagram shows the overlap between the three types of significant peaks detected within 10 kb of the TSS of annotated genes. The overlap regions represent genes with more than one type of peak within 10 kb of their TSS. 1) On only peak is when there is a significant peak in the Notch On sample but not in the Notch Off sample 2) Off only peak is when there is a peak in the Notch Off sample but not in the Notch On sample 3) Both peak is when there is a peak in both the Notch On and Notch Off Sample. Red is “on” and green is control. CBS is defined as CSL Binding Site.

To associate peaks with genes, a distance criteria from the transcriptional start site (TSS) was used ranging from +/− 20 Kb [67] to +/− 10 kb [68]. The Venn diagram in figure 3 indicates the number of genes with at least one peak within 10 kb. The “ON” only circle contains those genes that had a peak in cells expressing activated Notch and lacked a peak in the control cells. The “OFF” only circle are those genes that had a peak in the control cells and but not one in the cells expressing activated Notch. The “Both” circle indicates those genes that had a peak that was present in the activated Notch cells as well as the control cells. The cross-section indicates genes with multiple types of peaks. For example genes in the cross section of the “On” only circle and the “Off” only circle have at least one On only binding site and at least one Off only binding site. A total of 3077 genes had all three different types of peaks within 10 kb of their transcriptional start site. There were 540 genes that had CSL bound in normal cells but not in cells with constitutively active Notch.

Gene ontology analysis was performed to categorize the putative functions of genes contained in the “On” only category using Panther [47] to focus in on targets of overexpression of activated Notch1. Table 6 suggests crosstalk between Notch and several highly conserved pathways important in development at the level of transcription. Notch has been shown previously to integrate with these pathways including the PDGF signaling pathway (P-value = 4.44×10^−6^), ubiquitin proteasome pathway (P-value = 9.5×10^−6^), p53 pathway (P-value = 1.79×10^−5^), Ras pathway (P-value = 3.06×10^−4^), and cell cycle pathways (P-value = 1.32×10^−3^) in controlling development [47]. 18 out of 19 pathways were shown to crosstalk with Notch in previous studies (Table 6). A substantial number of genes do not have gene ontology annotations and are listed as unclassified. Finally, Table 7 shows the pathways sorted by number of genes in the pathway that are regulated by Notch and is used mainly as a classification tool. This table is to simply list the pathways represented by the genes with at least one On only peak. A substantial number of genes do not have gene ontology annotations and are listed as unclassified.

**Table 6:** Gene Ontology analysis of genes with at least one On only peak sorted by P-value.

Pathways	#	P value	Enrichment	
Cell cycle	23	0.00132	2.9	Regulates Cyclin D3 [69]
p53 pathway by glucose deprivation	22	0.00662	2.7	
p53 pathway feedback loops 2	39	0.00084	2.3	
Interferon-gamma signaling pathway	21	0.0983	2.3	regulates proliferation and IFN-gamma production [70]
Ubiquitin proteasome pathway	61	9.5E-06	2.2	Induces transcription of SKP2 [71]
Transcription regulation by bZIP TFs	40	0.00289	2.1	N/A
General transcription regulation	29	0.0344	2.1	
p53 pathway	74	1.8E-05	2.0	Suppresses p53 in Cancer [72], [73]
Ras Pathway	54	0.00031	2.0	Notch/Kras coactivation promotes reprogramming [74], [75], [76], [77]
Hedgehog signaling pathway	24	0.247	2.0	Cross talk in medulloblastoma [5], [78], [79]
Hypoxia response via HIF activation	20	0.609	2.0	HIF-1alpha interacts and acts in synergy with NICD at Notch targets [80], [81], [82]
VEGF signaling pathway	41	0.0326	1.9	Directly regulates of VEGFR-3 [83]
p38 MAPK pathway	33	0.0642	1.9	Suppresses the activity of p38 MAPK [84]
PDGF signaling pathway	96	4.4E-06	1.8	Regulates PDGFb in muscles [85], [86]
B cell activation	49	0.0125	1.8	B cell terminal differentiation and Marginal Zone B cells [87], [88], [89], [90]
FGF signaling pathway	66	0.0159	1.7	Suppresses FGF transformation [91]
Parkinson disease	59	0.025	1.7	
Toll receptor signaling pathway	35	0.335	1.7	Regulates Toll receptors and IL-6 [92], [93]
Oxidative stress response	36	0.535	1.7	
Alzheimer disease-amyloid secretase pathway	34	0.595	1.7	
FAS signaling pathway	22	1	1.7	
EGF receptor signaling pathway	70	0.013	1.6	Components are direct targets [16]
PI3 kinase pathway	54	0.135	1.6	Activates PI3 Kinase Pathway [94], [95]
Dopamine receptor mediated signaling pathway	38	0.48	1.6	Reduces dopaminergic spinal cord neurons [96]
Interleukin signaling pathway	70	0.185	1.5	Binds IL7R gene promoter [97]

**Table 7:** List of pathways sorted by number of Gene Ontology analysis of genes with at least one On only peak sort by number of genes.

	On Only		
Pathways	#	P value	Enrichment
Unclassified	7242	2.43E-09	0.97
Inflammation mediated by chemokine and cytokine signaling pathway	111	1	1.18
Wnt signaling pathway	107	1	0.94
Angiogenesis	97	0.477	1.35
PDGF signaling pathway	96	0.00000444	1.85
Integrin signalling pathway	93	1	1.27
Huntington disease	79	1	1.34
Apoptosis signaling pathway	76	0.194	1.45
p53 pathway	74	0.0000179	1.96
Interleukin signaling pathway	70	0.185	1.48
EGF receptor signaling pathway	70	0.013	1.64
FGF signaling pathway	66	0.0159	1.65
T cell activation	63	1	1.34
Ubiquitin proteasome pathway	61	0.0000095	2.16
TGF-beta signaling pathway	61	0.926	1.42
Parkinson disease	59	0.025	1.68
Ras Pathway	54	0.000306	2.03
PI3 kinase pathway	54	0.135	1.60
Heterotrimeric G-protein signaling -Gi alpha and Gs alpha mediated	54	1	1.09
B cell activation	49	0.0125	1.83
Cytoskeletal regulation by Rho GTPase	43	1	1.14
Heterotrimeric G-protein signaling -Gq alpha and Go alpha mediated	42	1	0.93
VEGF signaling pathway	41	0.0326	1.86
Endothelin signaling pathway	41	1	1.51
Transcription regulation by bZIP transcription factor	40	0.00289	2.11
p53 pathway feedback loops 2	39	0.000841	2.25
Alzheimer disease-presenilin pathway	39	1	0.98
Dopamine receptor mediated signaling pathway	38	0.48	1.64
Oxidative stress response	36	0.535	1.65
Insulin/IGF pathway-protein kinase B signaling cascade	36	1	1.45
Toll receptor signaling pathway	35	0.335	1.72
Alzheimer disease-amyloid secretase pathway	34	0.595	1.67
p38 MAPK pathway	33	0.0642	1.94
General transcription regulation	29	0.0344	2.12
Nicotinic acetylcholine receptor signaling pathway	25	1	0.86
Muscarinic acetylcholine receptor 1 and 3 signaling pathway	25	1	1.36
Metabotropic glutamate receptor group III pathway	25	1	1.08
5HT2 type receptor mediated signaling pathway	24	1	1.18
Hedgehog signaling pathway	24	0.247	2.00
Cadherin signaling pathway	24	0.000102	0.42
Thyrotropin-releasing hormone receptor signaling pathway	23	1	1.21
Insulin/IGF pathway-MAP kinase cascade	23	1	1.07
Cell cycle	23	0.00132	2.94
p53 pathway by glucose deprivation	22	0.00662	2.72
Oxytocin receptor mediated signaling pathway	22	1	1.23
Muscarinic acetylcholine receptor 2 and 4 signaling pathway	22	1	1.25
FAS signaling pathway	22	1	1.75
Angiotensin II- signaling through G proteins and beta-arrestin	21	1	1.37
Metabotropic glutamate receptor group II pathway	21	1	1.25
Interferon-gamma signaling pathway	21	0.0983	2.28
Axon guidance mediated by semaphorins	21	1	1.50
Beta2 adrenergic receptor signaling pathway	20	1	1.49
Hypoxia response via HIF activation	20	0.609	1.99
5HT1 type receptor mediated signaling pathway	20	1	1.43
Notch signaling pathway	19	1	1.31

Of the 148 Notch regulated genes identified by microarray and assessed by ChIP-chip, 59 were shown by ChIP-seq to be bound by activated Notch responsive CSL and 28 out of 54 (52%) were common to ChIP-chip and ChIP-seq (Table 8). Although 52% overlap was significant (P-value less than 0.02), ChIP-seq may be missing peaks because 1) the ChIP-chip approach would be more likely to identify weaker sites because of the custom array tiled each 1.5 kb region with a higher resolution (at least 30 probe duplicate probes per 1.5 kb region whereas ChIPseq unique reads mapped about 1 read per 1 kb), 2) peaks near repetitive regions would not be identified by ChIP-seq.

**Table 8:** Differentially regulated genes shown by ChIP-seq to be bound by activated Notch responsive CSL.

Affy ID	P-value	Foldchange	Common	Genbank	ChiPchip(P)
1438511_a_at	0.034759	3.541205714	1190002H23Rik	BB408123	1
1424968_at	0.042857	1.741077596		BC027185	0
**1427425_at**	0.018985	**2.555648905**	**9130208E07Rik**	**BC026435**	**na**
1420336_at	0.026182	2.092625895	2010109H09Rik	NM_025629	1
1425405_a_at	0.04146	0.471274144	Adar	AF291876	1
1422631_at	0.01076	4.516146778	Ahr	NM_013464	1
1418571_at	0.040063	6.114235523	Fn14-pending	NM_013749	1
1418204_s_at	0.049498	5.204132297	Aif1	NM_019467	1
1452747_at	0.033843	0.579953607	1110012E06Rik	BM944122	1
1419406_a_at	0.012028	1.750707165	Bcl11a	NM_016707	1
1450355_a_at	0.046005	0.555631942	Capg	NM_007599	1
1416401_at	0.003304	2.508508142	Kai1	NM_007656	1
1418328_at	0.016035	2.403499989	Cpt1b	AF017174	0
1448591_at	0.019113	2.227026837	Ctss	NM_021281	1
1438133_a_at	0.048322	2.23959795	Cyr61	BM202770	0
1428306_at	0.007377	2.544732022	5830413E08Rik	AK017926	0
1435493_at	0.049644	2.803243131	AA407887	AV297961	0
1435494_s_at	0.136552	2.476938896	AA407887	AV297961	0
1449222_at	0.02364	0.474559067	Ebi3	NM_015766	1
1460356_at	0.013887	2.351055383	Esam-pending	AF361882	0
1434348_at	0.011362	0.553337783	D17Ertd315e	BM206792	1
1416855_at	0.02831	3.084525151	Gas1	NM_008086	1
1418949_at	0.049119	2.313760679	Gdf15	NM_011819	1
1418350_at	0.035829	2.229833753	Hegfl	L07264	0
1418102_at	0.041252	2.126284827	Hes1	BC018375	1
1415999_at	0.001444	22.97309568	Hey1	NM_010423	1
1417014_at	0.043452	2.99304653	Cryac	AF250139	1
**1417013_at**	0.002819	**3.18788396**	**Cryac**	**NM_030704**	**na**
1424112_at	0.00868	1.984754433	Igf2r	BG092290	0
1420860_at	0.026274	6.660282574	Itga9	BG067332	1
1450029_s_at	0.113226	2.313648867	Itga9	BG067332	1
**1460285_at**	0.013047	**2.656757581**	**Itga9**	**NM_133721**	**na**
1421106_at	0.031982	2.920188923	Jag1	AA880220	0
1417395_at	0.049484	2.221262262	Klf4	BG069413	0
1448169_at	0.047632	4.658619972	Krt1-18	NM_010664	1
1449328_at	0.033057	2.032396003	Ly75	NM_013825	0
1426306_a_at	0.003296	2.440930191	Maged2	AF319976	0

### miRNA are differentially regulated during normal hematopoiesis

**Figure 4:**
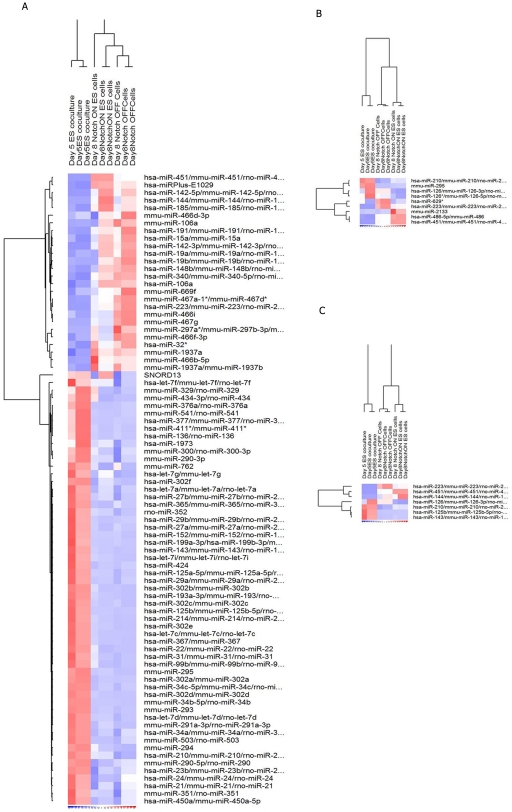
MicroRNA differentially regulated from Day 5 to Day 8. Dendrogram showing a) the 83 differentially regulated microRNAs from day 5 to day 8. b) microRNAs that were differentially regulated comparing Day 8 Notch ON to Notch OFF and c) the microRNAs used for real-time PCR analysis. Data analyses were performed by using DNA-Chip Analyzer 1.3 [117]. The thresholds for selecting significant genes were set at a relative difference of >1.5-fold, an absolute difference of >100 signal intensity, and P<0.05.

MicroRNA are important regulator of hematopoiesis and we wanted to assess which microRNA are differentially expressed during hematopoiesis in our in vitro murine embryonic stem cell co-culture system. Expression profiles of miRNA during normal hematopoietic differention were examined using a commercial miRNA microarray that contained all miRNA present in the miRBase miRNA registry release 8.1 [98], [99]. To assess if miRNA are differentially regulated during normal hematopoiesis, the expression profile of miRNA from hematopoietic progenitors (day 8) were compared to flk1+ hemangioblasts (day 5). Each time point was represented by two independent biological samples and two technical replicates. Microarray analysis revealed that 83 miRNAs were significantly differentially expressed by greater than 1.5-fold during the course of hematopoiesis *in vitro* from day 5 to day 8 (table S1). Thirty one miRNA were up-regulated and fifty two were down-regulated. The differentially regulated miRNA cluster into groups (see figure 4a).

### Analysis of activated Notch 1 responsive CSL binding to miRNA loci

To assess if miRNA expression was influence by the Notch pathway, CSL binding sites were mapping to within 2 kb of either the genomic loci or the promoter region of all known miRNA. We assessed if the ChIPseq derived CSL binding site were mapping in the high-confidence microRNA promoters described by the Young lab which represented over 80% of miRNA [100]. There were 37 miRNA with at least one binding site within 2 kb of their transcription start site (Table S2). There were several binding sites that were mapped onto the start of miRNAs (data not shown).

### Overexpression of Notch 1 alters miRNA expression during hematopoiesis

**Figure 5:**
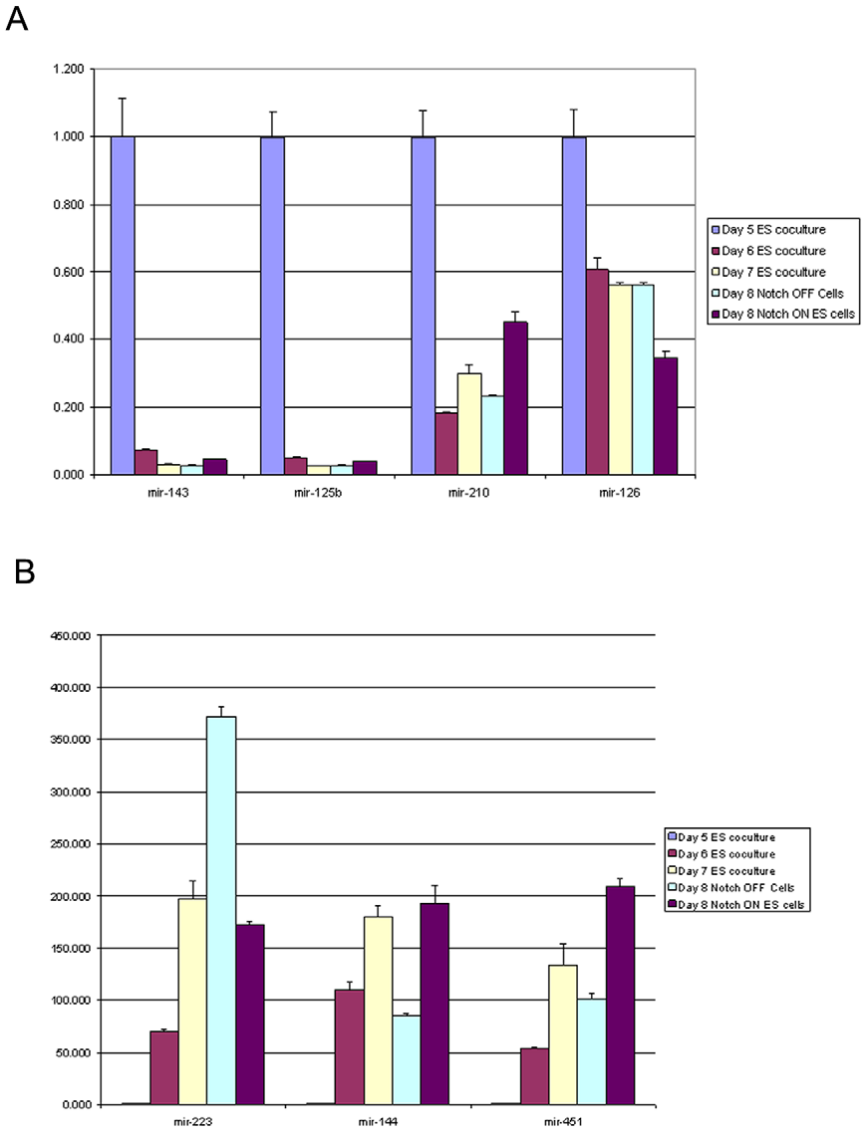
Seven MicroRNA expression on day 5, 6, 7 and 8. Seven mature microRNAs including a) four downregulated and b) three upregulated that whose expression was analyzed using Taqman miRNA expression assay on day 5,6,7 and 8. The expression of the miRNA was normalized against the expression level of the control miRNA snoRNA202 (AF357327) and presented as the mean normalized expression.

Since, the CSL binding sites were mapping to the promoter of miRNA and close to their genomic loci, we wanted to assess if overexpression of Notch 1 was influencing miRNA expression. Comparing the expression profile of miRNA from normal hematopoietic progenitors (day8-Notch Off) to cells with overexpression of activated Notch 1 (day8-Notch On) microarray analysis showed that 10 genes were differentially expressed by greater than 1.5 fold (Table 9). Four were down regulates (Figure 5a) and six miRNAs were up-regulated (Figure 5b) as a result of activated Notch1 overexpression from day 5 to day 8.

**Table 9:** miRNAs differentially regulated by overexpression of ligand independent Notch1.

probe set	Day 8 Notch OFF	Day 8 Notch ON	Day 8 Notch ON versus Notch OFF	P value
mmu-miR-210	132.77	306.83	2.31	0.0083
mmu-miR-451	16123.4	35723.37	2.22	0.0258
mmu-miR-486	69.59	142.45	2.05	0.0365
mmu-miR-125b-5p	187.47	382.53	2.04	0.0506
mmu-miR-2133	785.97	1450.26	1.85	0.0255
mmu-miR-295	91.52	163.06	1.78	0.0163
mmu-miR-126-5p	386.06	255.75	−1.51	0.0044
mmu-miR-126-3p	2791.95	1816.94	−1.54	0.0063
hsa-miR-629*	151.56	83.97	−1.8	0.0048
mmu-miR-223	3566.25	1928.69	−1.85	0.0162

### MiRNA expression profile of cells with Notch 1 overexpression mimic miRNA expression of cells in a less mature state

To further confirm the miRNA differentially regulated during normal hematopoiesis, we selected seven miRNA and used RT-PCR Taqman assays, to assess their expression on day 5, 6, 7 and 8. These seven miRNA represented four clusters (see figure 4b and 4c). Cluster I are miRNA whose expression is upregulated during normal hematopoiesis and further upregulated with overexpression of Notch 1. Cluster II, are miRNA that are also upregulated during normal hematopoiesis but their expression is attentuated with the overexpression of Notch 1. Cluster III and IV represent miRNA whose expression are down regulated during normal hematopoiesis but the overexpression of Notch 1 either further upregulates their expression (Cluster III) or attenuates their expression (cluster IV). Of the seven, microarray analysis showed that four were down-regulated and three were upregulated (see figure 5a and 5b). The expression of all seven miRNA as assessed by the Taqman assays corroborated the microarray assay (see Table 10 and 11) in their expression patterns. Furthermore, analysis of miRNA expression during day 6 and 7 in addition to day 5 and day 8 revealed the kinetics of their expression. For the down-regulated miRNAs, miR-143 and miR-126b were down regulated by at least ten fold by day 6. MiR 143 was downregulated from 10 fold on day 6 to 33 fold by day 7 and then 35 fold by day 8. Mir-125b was downregulated from 20 fold on day 6 to 38 fold on day and 33 fold on day 8. Both miR-210 and miR-126 were downregulated less dramatically from day 5 to day 8 with miR-210 levels experiencing its most dramatic change at day 7 and miR-126 at day 8 even though there was little difference in the downregulation between day 6 (−1.65), day 7 (−1.780) and day 8 (−1.785). Among the three up-regulated miRNA, miR-223 progressively increased from day 5 to day 8 whereas miR-144 and miR-451 peaked at day 7.

**Table 10:** miRNA differentially regulated during normal hematopoiesis and affected by the overexpression of activated Notch.

probe set	Day 5	Day 8 Notch OFF	Day 8 Notch ON	Day 8 Notch OFF versus Day 5	P value	Day 8 Notch ON versus Notch OFF	P value
mmu-miR-451	141.46	16123.4	35723.37	113.98	0.0024	2.22	0.0258
mmu-miR-223	24.31	3566.25	1928.69	146.73	0.0071	−1.85	0.0162
mmu-miR-144	36.83	2409.56	4770.81	65.42	0.0489	1.98	0.2094
mmu-miR-126-3p	3155.6	2791.95	1816.94	−1.13	0.5520	−1.54	0.0063
mmu-miR-210	573.25	132.77	306.83	−4.32	0.0099	2.31	0.0083
mmu-miR-143	2071.2	39.64	103.9	−52.25	0.0170	2.62	0.0637
mmu-miR-125b-5p	7859.6	187.47	382.53	−41.93	0.0483	2.04	0.0506

**Table 11:** Timecourse of miRNA expression from Day 5 to Day 8 and the effect of overexpression of activated Notch.

	Day 5	Day 6	Day 7	Day 8 Notch OFF	Day 8 Notch ON
mir-223	1.00	70.21	197.62	372.17	172.26
mir-451	1.00	53.59	133.43	101.39	209.60
mir-144	1.01	110.24	180.22	84.87	193.33
mir-126	1.00	−1.65	−1.78	−1.79	−2.91
mir-210	1.00	−5.47	−3.37	−4.30	−2.22
mir-125b	1.00	−20.03	−38.33	−35.60	−26.03
mir-143	1.00	−13.63	−33.35	−36.54	−22.13

We have shown that Notch 1 overexpression is associated with keeping cells in a less differentiated state and the miRNA profiles corroborate this claim. Notch 1 overexpression is effecting the timing of miRNA expression during hematopoiesis. When ligand independent Notch 1 is introduced at day 5, the miRNA expression is delayed. miR-223 and miR-144 day 8 Notch ON fold change levels (172 and 193 respectively) mimic day 7 levels (197 and 180 respectively). For the down-regulated miRNA, their down regulation was attenuated with overexpression of Notch 1.

## Discussion

The identification of multiple sites with P-values less than 10^−12^ and the fact that reads were preferentially mapped close to the TSS indicated that we were likely sequencing true binding sites in the Chip-Seq experiments. Nonetheless the number of genes that were shown to be bound by CSL (10,327) seems to be higher than expected. However, the Gene Ontology analysis indicated that CSL binding sites are preferentially concentrated in important signaling pathways which have previously been shown to interact with the Notch pathway (see Table S1). Therefore, it is our hypothesis that these large numbers of genes bound by Notch are in fact likely true targets. In previous studies that used ChIPseq to identify transcription factor binding sites genome wide, the number of binding sites identified ranged from 1858 to over 60,000 [101]. For STAT1 there were over 40,000 sites identified in stimulated Hela S3 cells while there were over 11,000 sites in unstimulated cells [102]. There were over 11,000 Fox 2a binding sites identified in adult liver [68]. The number of genes associated with transcription factor ranged from 1513 to 8411 in a comprehensive ChIPseq study that mapped 13 transcription factors in mouse ES cells [101].

The presence of CSL upstream of many genes involved in signaling pathways indicates that crosstalk between Notch signaling and other pathways may be at the level of transcription. It is unlikely that genes of important signaling pathways are controlled by a single transcription factor. Highly regulated genes are more likely to be controlled combinatorially by many transcription factors. This could explain the seemingly contradictory roles that Notch plays in cancer. In some cells, Notch functions as an oncogene [9] when aberrantly expressed. In other cell contexts it can function as a tumor suppressor [5]. It is not the presence of the CSL that solely determines the transcriptional state of its target genes, but combinations of transcription factors that converge on a regulatory region collectively that control the transcription of the gene and hence the transcriptome of a cell.

There were 540 genes that had CSL bound in normal cells but not in cells with constitutively active Notch. There are two explanations for this observation. First, CSL dependent Notch signaling maybe involved in the repression of genes. This observation would explain a novel mechanism for CSL dependent Notch signaling. Second, it is possible that the effects of NICD overexpression could be merely a result of NICD titrating CSL away from other binding partners [103]. Since constitutively active Notch is associated with oncogenesis and CSL binding is so prevalent through out the genome, a possible mechanism of oncogenesis would be disrupting the homeostasis of CSL binding.

Although the identification of differentially regulated genes by microarray analysis is important to understand transcriptional networks, it provides only limited information on the regulation of genes. First, microarray analysis cannot distinguish between direct and indirect targets. The presence of a CSL binding site in the promoter of a gene can be indicative of direct regulation by Notch signaling. CSL binding sites were observed within a region 1.5 kb of the TSS of several pathways known to crosstalk with Notch such as Wnt signaling [104] (during hematopoiesis), integrins [105] and the cadherins [95], and our ChIP-seq study provides the first evidence that Notch regulates these pathways directly.

Second, microarray analysis can not identify genes that are essential mediators of transcriptional regulation unless they are differentially regulated. The CSL transcription factor is a prime example. It was not among the 158 differentially regulated genes because Notch does not affect its transcription. However, it is the main transcription factor mediating Notch regulation. The potential utility of motif profiling can be highlighted by the fact that 12 out of 13 transcription factors, including CSL, that were identified by motif profiling and may be important in mediating Notch regulation were not seen by microarray analysis because their expression levels do not change.

Although we cannot conclude from an *in silico* method such as CLOVER that the CSL binding sites are actually bound *in vivo*, we can say they represent putative sites that can be bound by CSL. The success rate of picking bona fide binding sites using *in silico* methods is dependent on the characteristics of the transcription factor. Some of the latest ChIP-chip findings indicate that these *in vitro* derived DNA binding sites may not necessarily represent all the binding sites of the proteins *in vivo*. For example, two independent androgen receptor (AR) ChIP-chip studies showed that 90% of AR-binding sites did not contain the consensus ARE motif [106]. Similarly, 90–96% of ChIP-chip identified binding sites did not contain the *in vitro* derived E2F consensus [107]. However, other transcription factors show a converse correlation. Only 20% of the hepatocyte nuclear factor 4a (HNF4a) binding sites, 10% of the repressor element 1 (REST) binding sites lacked sequences resembling their respective *in vitro* derived consensus sequences [67]. Only 10 out of the 28 genes shown by CLOVER to have a putative CSL binding site were shown to be bound in vivo using ChIPseq analysis. Although motif finding algorithms can be used to assess binding site *in silico*, they may be limited to a particular type of binding sites and not the total breadth of binding sites.

To assess whether the putative CSL binding sites were actually bound *in vivo*, we devised a custom ChIP-seq approach which included a two step IP (Re-ChIP) to capture activated Notch responsive CSL binding site.

A single IP against CSL will not detect activated Notch- responsive CSL bound genes because the occupancy of the CSL binding site does not change in responsive to Notch activation [1]. Therefore, there will be little or no difference comparing CSL ChIP purified DNA from normal cells to cells with constitutively active Notch. There are several technical reasons why the two step IP approach is more likely to capture activated Notch responsive CSL binding sites. First CSL is a small protein (60 Kd) which is bound to DNA and is associated with very large repressor complexes. The crosslinking by formaldehyde makes the CSL epitope inaccessible by the CSL antibody and therefore the IP with antibody against CSL is weak (personal communication from J. Aster AACR 2008). Furthermore, CSL has been detected in the cytoplasm and it has been proposed by Krejci et al [63] that the interaction of the CSL activator complex with the binding site might be dynamic, meaning there is unbound CSL in the nucleus as well. Both these considerations imply that, in addition to the epitope being inaccessible, the antibody is being titrated by unbound CSL. By incorporating an IP step against acetylated histone H4 (or H3), we created a lysate that will maximize the likelihood that the CSL antibody will recognize the DNA bound CSL. The unbound CSL is washed away in the first IP and the chromatin complexity is reduced to sites that have acetylation at H4. Thus, we are using biological insight to improve sensitivity.

Coupling our Re-ChIP approach with next-generation sequencing technologies allowed us to assess genome-wide Notch responsive CSL binding sites genome-wide. We observed that the CSL binding sites were preferentially mapping within regions 1.5 kb upstream of TSS of genes, with the highest density of binding sites within 300 bp of the TSS. This indicated that the sequences that were being pulled down with our Re-ChIP procedure were not mapping randomly throughout the genome. Furthermore, comparison with the literature showed that CSL binding relative to the TSS was consistent with the binding site location of other important developmental transcription factors. Koudritsky [108] et al. used ChIP-chip data from nine human transcription factors (OCT4, SOX2, NANOG, HNF1A, HNF4A, HNF6, FOXA2, USF1 and CREB1) to show that they bind preferentially to proximal regions to the TSS and with strong binding within 300 bp of the TSS.

Finally, there seems to be a relation between microRNA and Notch signaling, although thus far, that relationship has been studied in regards to how microRNA target Notch1 or its targets. Here we investigated the alternative by looking which microRNA may be regulated by Notch1. First, we showed that there were over 30 microRNA with a CSL binding site within 2 kb of their transcription start site. That was the impetus for us to profile microRNA expression in our coculture system. The overexpression of ligand independent NICD resulted in the differential expression of 10 microRNAs of which six were upregulated and four were down regulated. miR-144/miR-451 and miR143 were most interesting. Both had a CSL binding site within 2 kb of their transcription start site indicating they maybe direct target and overexpression of NICD increased both of their expression. Mir-144 levels were below detection (<100) on the microarray on Day 5 and dramatically increased to 2400 by day 8 during normal hematopoiesis and its levels reached 4770 with the overexpression of NICD. Mir-143 levels were highest on day 5 at 2071 and by day 8 its expression levels had decrease to 187 during normal hematpoiesis and that decrease was attenuated by the overexpression of NICD. Further studies need to be done if the abberrent gene regulation caused by overexpression of ligand independent Notch may be abrogated by controlling the expression of either miRNA.

## Materials and Methods

### Motif Analysis

The CLOVER algorithm (Http://zlab.Bu.edu/clover/) was used to screen a target set of sequences against a motif library to determine over and under-represented motifs. For the target set, 1.5 kb upstream of transcriptional start site (TSS) sequences of the Notch regulated genes reported in Ganapati et. [45] were obtained from the UCSC genome database [109], [110] (http://genome.ucsc.edu/.). Motifs included the JASPAR motif library [111] (n = 123) along with a CSL motif [112]. Number of genes are synomous with number of Genbank accession number. Therefore, two Genbank accession number with one common gene name would be considered two genes.

Background sequences were used for determining statistical significance. The background included both Mouse Chromosome 19 and 2 kb upstream of all mouse genes. Mouse chromosome 19 is 42.8% C+G from NCBI Build 30, sequences 2000 bp upstream of mouse genes are 47.8% C+G from UCSC [109], [110]. The P value indicates the probability that the observed over- or under-representation of a motif is achieved by random selection and was determined by comparison to mouse chromosome 19 sequence and 2 kb upstream of all mouse genes.

### Cell Culture

ES cell *in vitro* differentiation and induction of truncated Notch1 expression were as previously described. Briefly, 22.5×10^4^ undifferentiated ES cells (E14Tg2a ES clone ZEDN1[45]) repressed for ZEDN1 expression were co-cultured on a confluent layer of OP9 stromal cells in a 225-cm flask in the presence of 100 ng/ml Tet. On day 5 of co-culture, both differentiated ES cells and the OP9 stromal cells[113] were harvested in fresh medium. Cells were re-plated in new flask for 20–30 minutes to separate OP9 cells from ES cells. OP9 cells quickly adhered to the dish, and ES cells were harvested. Flow cytometry analysis (stained with anti-Flk-1-PE, CD34-FITC, and CD117-PE (BD Pharmingen, San Diego, http://www.bdbiosciences.com/pharmingen) was performed on the ES cells harvested from the co-culture on Day 5 to assure that ES cells were differentiating as previously reported.

Then, 8×10^6^ day 5 ES cells were re-plated on 225-cm flask of confluent OP9 cells. These day 5 co-cultures were continued in both Notch-Off (Tet-On) and Notch-On (Tet-Off) conditions until day 8 when hematopoietic progenitors were harvested for flow analysis and ChIP assay. Flow analysis [anti-CD117-TC (c-Kit), anti-CD11b-TC, anti-CD34-PE (Caltag, Burlingame, CA, http://www.caltag.com), and anti-Ter119-PE (BD Pharmingen)] was done to ensure that the cells were differentiated as previously reported. Three separate experiments were performed.

### Re-ChIP

ChIP assays were performed as described by using the chromatin immunoprecipitation assay kit (Upstate Biotechnology, Waltham, MA) following the protocol supplied by the manufacturer with a few modifications including a second IP step. Briefly, 24×10^6^ day 8 hematopoietic progenitors harvested from the co-culture were cross-linked in 1% formaldehyde for 10 min at room temperature, collected, washed and spun down. The pellets were lysed in SDS lysis buffer at room temperature and sonicated for four 15 sec pulses (model 300; Fisher Sonic Dismembrator) to generate DNA with an average length of 0.5–1 kb. The nuclear lysate was diluted (dilution buffer) and pre-cleared with 100 ul salmon sperm/ protein A for 2 hrs at 4°C. Supernatants were incubated with anti-acetyl H4 IgG (Upstate Biotechnology, Waltham, MA) for 8 hrs at 4°C. Then 80 ul salmon sperm/ protein A were added and incubated for 2 hrs at 4°C. After extensive washes, and elution (1% SDS, 50 mM NaHCO3), the eluant was diluted 10X with PBS and incubated with anti-CSL IgG [114] for 8 hr at 4°C for a second IP. The immunoprecipitation step, washes and elution were repeated for the second IP. The eluants were then incubated at 65°C for 5 hr, followed by proteinase K treatment for 1 hr at 45°C. DNA fragments were purified by a chloroform/phenol extraction and an ethanol precipitation, resuspended in water, and stored at -20°C.

### Custom array ChIP-chip

The DNA was then prepared for hybridization according to the manufacture's procedure (Combimatrix, Mukilteo, WA). First DNA from Notch ON and Notch Off cells were amplified using Ligation Mediated PCR (LMPCR). Then the DNA was labeled with Cy5 and Cy3 fluorescent dyes respectively. The labeled DNA fragments were then used to probe custom tiling (tiled every 50 bp) arrays (Combimatrix, Mukilteo, WA) containing 4880 unique probes representing 1.5 kb promoter sequences of Notch regulated genes as well as background regions. The probes on the array are repeated at least once to cover the 12K probe custom array.

### Custom array analysis

For normalization, each probe was divided by the medium Cy5 (or Cy3) signal for the array. Identical probes on an array with a CV of greater than 0.3 were excluded in the analysis. Since three independent experiments were performed, the values for each probe across the three arrays were averaged for a given experimental condition. For each experiment, there were two arrays hybridized. One array was hybridized with ChIP purified DNA from anti-CSL (single IP) and the other array was hybridized with ChIP purified DNA from 2 cycle IP (anti-acetyl H4 IP followed by anti-CSL IP). The average values for each probe were used to determine enrichment. For each probe, values from the Notch On condition (activated Notch) were divided by values from the Notch Off condition (control cells) to determine a ratio. The log 2 ratio of Notch On to Notch Off was used to determine binding. ChIPOTle analysis [115], which identifies peaks using a sliding window approach, was performed for each array . The window was set to the average shear length of the DNA (250 bp) and the step was set to the probe size (50 bp). We used the Gaussian distribution to model the background or non-enriched population because it is the most powerful approach in ChIPOTLe for estimating the P-value for enrichment. It assumed the background to a symmetric Gaussian distribution about the mean of zero. The P-values reported by ChIPOTle are corrected for multiple comparisons using the conservative Bonferroni correction. Under the null hypothesis, the distribution of the average log2 ratio within each window is again Gaussian, with mean zero and Variance equal to the variance of a single log ratio divided by the number of elements in the window. Thus the nominal P-value for a window with average ratio *w* can be calculated using the standard error function (ERF) as follows: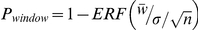 where σ is the standard deviation for the background distribution, and n is the number of microarray elements used in the window.

### ChIP-seq

ChIP-seq libraries were made from the purified Re-ChIP DNA fragments using the Illumina ChIP-seq Sample Prep Kit following the manufactures procedure. After the Re-ChIP DNA fragments were end-repaired, Illumina adapters were ligated to amplify the DNA. The amplified DNA was run on a 2% gel and fragments 150–300 bp in size were purified and sequenced on the Illumina Genome Analyzer according to the manufacturer's instruction. The resulting flow-cell was sequenced for 36 cycles to generate 36-bp reads. The sequencing was performed using the Jonsson Cancer Center Gene Expression Shared Resource (http://www.cancer.ucla.edu/Index.aspx?page=150).

The Eland alignment tool was used to align the first 32 bp of every read to the mouse reference genome (NCBI build 37, mm9) allowing for 2 base mismatches per sequence. Only uniquely mapped reads were considered for further analysis.

Uniquely-aligned sequences (reads) were then subjected to a peak analysis algorithm which counts all reads within a given 1000 base window relative to genomic positions. A step size of 50 bases was used for window overlap. The Poisson distribution [116] was used to generate P-values for each 1000 base window using the observed and expected counts (the average number of reads in a 1 kb window in the genome) and windows were filtered by these values to generate a list of peaks.

Peaks were defined by several criteria: (a) a window P-value less than 10^−12^, (b) a Notch On/Notch Off difference greater than 2 and (c) a window mapability greater than 25% (i.e. more than 25% of the 32mers in the window were unique to the genome).

This list of peak positions was then filtered by their genomic positions relative to +/− 10 kb of all known TSS (Fig. 4.1). The same process was repeated using an equal number of randomly sampled 32 base sequences as a control to determine the false discovery rate of the peak-finding algorithm (data not shown), which was less than 1%.

### miRNA Microarray and Data Analysis

Total RNAs were isolated by TRIzol (Invitrogen) from the ES/OP9 coculture at day 5, dau 8 Notch-On and Day 8 Notch Off. The labeled miRNAs were hybridized to miRCURY™ locked-nucleic acid array version 8.1 (Exiqon) according to the instructions of the manufacturer. Data analyses were performed by using DNA-Chip Analyzer 1.3 [117]. The thresholds for selecting significant genes were set at a relative difference of >1.5-fold, an absolute difference of >100 signal intensity, and P<0.05. All data is MIAME compliant and the raw data has been deposited in Gene Expression Omnibus (GEO) (GEO Accession # GSE28338), a MIAME compliant database.

### Taqman® miRNA Expression Assays

RNA was reverse-transcribed using specific miRNA stem-loop primers [118] and the Taqman® miRNA reverse transcription kit (Applied Biosystems). Mature miRNA expression was measured with Taqman® microRNA assays (Applied Biosystems) according to the manufacturer's instructions. The expression of the miRNA was normalized against the expression level of the control miRNA snoRNA202 (AF357327) and presented as the mean normalized expression.

## Supporting Information

Table S1 Differentially regulated miRNA during normal hematopoiesis.
(XLS)

Table S2 CSL binding sites within 2 kb of the TSS of microRNAs.
(XLS)
